# Serum lactate is associated with increased illness severity in immunocompromised pediatric hematology oncology patients presenting to the emergency department with fever

**DOI:** 10.3389/fonc.2022.990279

**Published:** 2022-10-06

**Authors:** Leonora Rose Slatnick, Kristen Miller, Halden F. Scott, Michele Loi, Adam J. Esbenshade, Anna Franklin, Alisa B. Lee-Sherick

**Affiliations:** ^1^ Department of Pediatrics, Center for Cancer and Blood Disorders, University of Colorado Anschutz Medical Center, Children’s Hospital Colorado, Aurora, CO, United States; ^2^ Department of Pediatrics, Section of Pediatric Emergency Medicine, University of Colorado Anschutz Medical Center, Children’s Hospital Colorado, Aurora, CO, United States; ^3^ Department of Pediatrics, Division of Critical Care Medicine, University of Colorado Anschutz Medical Center, Children’s Hospital Colorado, Aurora, CO, United States; ^4^ Department of Pediatrics, Vanderbilt University Medical Center and Vanderbilt Ingram Cancer Center, Nashville, TN, United States

**Keywords:** lactate, pediatric oncology, sepsis, serious bacterial infection, immunocompromised, chemotherapy-related immunosuppression, clinical deterioration

## Abstract

**Introduction:**

Determining which febrile pediatric hematology/oncology (PHO) patients will decompensate from severe infection is a significant challenge. Serum lactate is a well-established marker of illness severity in general adult and pediatric populations, however its utility in PHO patients is unclear given that chemotherapy, organ dysfunction, and cancer itself can alter lactate metabolism. In this retrospective analysis, we studied the association of initial serum lactate in febrile immunosuppressed PHO patients with illness severity, defined by the incidence of clinical deterioration events (CDE) and invasive bacterial infection (IBI) within 48 hours.

**Methods:**

Receiver operating characteristic (ROC) curves were reported using initial lactate within two hours of arrival as the sole predictor for CDE and IBI within 48 hours. Using a generalized estimating equations (GEE) approach, the association of lactate with CDE and IBI within 48 hours was tested in univariate and multivariable analyses including covariates based on Quasi-likelihood under Independence Model Criterion (QIC). Additionally, the association of lactate with secondary outcomes (i.e., hospital length of stay (LOS), intensive care unit (PICU) admission, PICU LOS, non-invasive infection) was assessed.

**Results:**

Among 897 encounters, 48 encounters had ≥1 CDE (5%), and 96 had ≥1 IBI (11%) within 48 hours. Elevated lactate was associated with increased CDE in univariate (OR 1.77, 95%CI: 1.48-2.12, p<0.001) and multivariable (OR 1.82, 95%CI: 1.43-2.32, p<0.001) analyses, longer hospitalization (OR 1.15, 95%CI: 1.07-1.24, p<0.001), increased PICU admission (OR 1.68, 95%CI: 1.41-2.0, p<0.001), and longer PICU LOS (OR 1.21, 95%CI: 1.04-1.4, p=0.01). Elevated lactate was associated with increased IBI in univariate (OR 1.40, 95%CI: 1.16-1.69, p<0.001) and multivariable (OR 1.49, 95%CI: 1.23-1.79, p<0.001) analyses. Lactate level was not significantly associated with increased odds of non-invasive infection (p=0.09). The QIC of the model was superior with lactate included for both CDE (305 vs. 325) and IBI (563 vs. 579).

**Conclusions:**

These data demonstrated an independent association of elevated initial lactate level and increased illness severity in febrile PHO patients, suggesting that serum lactate could be incorporated into future risk stratification strategies for this population.

## Introduction

Infectious complications in the setting of therapy-related immunosuppression are a significant cause of morbidity and mortality in pediatric hematology/oncology (PHO) patients. Due to the risk of rapid clinical deterioration from bacterial infection in this population (1–3), patients with fever who are categorized as high risk due to neutropenia (absolute neutrophil count <500 cells/mm^3^, or <0.5 x10^3^/μL) are often started on empiric broad spectrum intravenous (IV) antibiotics. Although the majority of these patients remain clinically well without an identifiable source of fever (4, 5), a subset of febrile PHO patients will decompensate despite empiric antimicrobial administration, with an associated mortality of 12-30% in those who progress to sepsis or septic shock (1, 6, 7). Timely recognition and treatment of septic shock is associated with reduced mortality and organ dysfunction. Thus, tools that enhance early detection of patients at greatest risk for progression to septic shock has potential to improve patient outcomes (8, 9). The ability to distinguish which patients will clinically deteriorate due to sepsis is challenging given the lack of effective reliable tools to risk stratify febrile PHO patients.

There remains a critical need in this population to optimize strategies that improve the ability to recognize which febrile patients require immediate intervention and identify patients whose antimicrobial therapy can be safely withheld or de-escalated. The PHO patient population presents unique challenges when it comes to the development of risk stratification tools as patients often lack the clinical signs and symptoms of severe infection at initial fever presentation due to an insufficient immune response (10–12). Furthermore, laboratory markers that are useful in distinguishing septic from non-septic patients in general pediatric and adult populations have questionable reliability in PHO patients who have altered baseline metabolism, immune capabilities, and organ function (13–17). For instance, previous studies evaluating the utility of c-reactive protein (CRP), procalcitonin (PCT), and inflammatory cytokines in this population have yielded conflicting results, thus no reliable biomarker has been established (11, 18–23).

The absolute neutrophil count (ANC) at the time of febrile presentation is a widely incorporated prognostic laboratory value used in PHO patients, typically characterized by the presence or absence of neutropenia, which is often incorporated into institutional clinical management guidelines in terms of antimicrobial administration and need for inpatient hospital admission. Although the risk of invasive infection is higher in this group compared to the general pediatric population (24–26), it is difficult to identify exactly which febrile patients with neutropenia have an active infection and which patients will go on to clinically deteriorate. Furthermore, severe infection can still develop in patients with adequate neutrophil counts, and the widespread incorporation of immune stimulating drugs into cancer therapy regimens may cloud the reliability of ANC as a prognostic indicator of poor infectious outcomes.

Lactate, a byproduct of tissue hypoperfusion, is one of the most extensively studied biomarkers for sepsis in adult and pediatric patients (27–32), and elevated serum lactate levels are associated with poor outcomes even in the setting of maintained oxygenation and arterial blood pressure (33, 34). It is well-established that patients with malignancy have altered lactate metabolism, as evidenced by presence of lactic acidosis in patients with malignancy in the absence of infection (35–37), and chemotherapy-related fluctuations in levels of serum lactate and lactate dehydrogenase (LDH), an enzyme which catalyzes the interconversion of pyruvate and lactate (38–40). Furthermore, many chemotherapeutic agents and immunosuppressive therapies can affect liver and kidney function, which play an essential role in lactate clearance (41, 42). Although studies in broad pediatric populations which include patients with chronic comorbidities have demonstrated an association of increased lactate with organ dysfunction, pediatric intensive care unit (PICU) admission, bacterial infection, and mortality (34, 43–45), there is a paucity of data regarding the discriminatory value of serum lactate in PHO patients explicitly.

A systematic review of 37 studies evaluating 24 different biomarkers in pediatric patients with fever and neutropenia by Haeusler et al. reported extensive evaluation of CRP (n=17 studies), PCT (n=9 studies), and several cytokines, most commonly IL-6 and IL-8 (46). Conversely, the literature regarding serum lactate in PHO patients is limited to a study performed by Pacheco-Rosas et al. at the Hospital de Pediatría del CMN Siglo XXI, which demonstrated an association (81% sensitivity, 83% specificity) between serum lactate level ≥2 mmol/L obtained within 48 hours of admission and severe sepsis in 100 pediatric oncology patients with fever and neutropenia (47), and a study performed in Thailand by Suwanpakdee et al. which reported an association between initial serum lactate >2.5 mmol/L with septic shock in 100 hemodynamically stable pediatric oncology patients with fever and neutropenia (ROC area 0.90, 95% CI: 0.81, 0.98) (48). Both studies suggest that there is a role for measuring serum lactate in this patient population, however generalizability is limited by small sample size, exclusion of non-neutropenic patients, and variable time allotted for initial serum lactate collection.

Identification of patients at high risk of sepsis or septic shock prior to progression of their symptoms is essential for early diagnosis and prompt resuscitation, the most efficacious strategy for preventing clinical decompensation, organ failure, and/or death (8, 9, 49–53). The objective of this study is to better understand the implications of lactate levels in febrile PHO patients by determining the association between initial venous lactate level and poor clinical outcomes, including clinical deterioration events (CDE) and invasive bacterial infection (IBI).

## Methods

### Data source

This single-center, observational study utilized the Colorado Sepsis Treatment and Recognition Registry, a database approved by the Children’s Hospital Colorado (CHCO) Organization Research Risk and Quality Improvement Review Panel and the Colorado Multiple Institution Review Board, which contains retrospectively collected data extracted from the electronic medical record (EMR) from pediatric Emergency Department (ED) encounters with ED clinician concern for possible sepsis as described by Scott et al (43). The registry includes ED encounter data for pediatric patients who are identified as high risk for sepsis, including patients with underlying oncologic or hematologic disorders who presented with fever or concern for infection. Relevant data that was not included in the registry was extracted from the EMR.

Encounters among immunocompromised PHO patients 0-25 years of age who underwent evaluation for fever in the CHCO ED between May 2012 and February 2019 were eligible for inclusion. This institution defines fever as a single temperature ≥101°F or two temperatures ≥100°F within a 24-hour period separated by at least two hours. PHO patients were considered immunocompromised if they were being treated with chemotherapy or were within six months of therapy completion, had a hematologic disorder requiring immunosuppressive therapy, or underwent hematopoietic stem cell transplantation (HSCT) within the previous six months. Encounters during which the patient was diagnosed with a new oncologic process were also included. Encounters were excluded if the patient had a known metabolic disorder, was transferred from an outside medical facility, arrived *via* emergency medical services (EMS) transport, or if a venous lactate level was not assessed within two hours of ED arrival. Multiple encounters per patient could be included, however encounters were excluded if the patient had been evaluated for fever/infection within the previous 72 hours.

Encounters among patients who were critically ill appearing upon ED presentation were considered separately, characterized by one or more of the following criteria: systolic hypotension <5^th^ percentile for age (54) on intake vital sign assessment, occurrence of at least one (≥1) CDE qualifying event (defined below) or PICU transfer within two hours of ED arrival, or provider documentation of critical appearance on initial assessment. Clinical data and outcomes for these encounters are briefly described in the results section, but were otherwise not incorporated into the analysis, as the goal of this study was to evaluate the association of lactate level with poor outcomes in patients whose illness severity was not immediately apparent upon initial presentation.

### Variables and outcomes

We tested the association of initial venous lactate level (mmol/L) obtained within two hours of ED arrival (primary variable) and covariates with the occurrence of CDE, IBI, and secondary outcomes pertaining to illness severity. Per institutional standard practice, a serum lactate level is obtained in conjunction with a complete blood count and blood cultures from all PHO patients who present to the ED with fever. Covariates were selected *a priori* based on clinical relevance for PHO patients and other established sepsis risk factors, including patient characteristics (i.e., age, underlying diagnosis, chemotherapy regimen intensity, phase of therapy, central venous access), encounter-specific variables assessed within two hours of ED arrival (i.e., WBC counts, presence of vital sign abnormalities), and patient- or provider-reported symptoms noted in the EMR (i.e., upper respiratory infection (URI) symptoms, chills and/or rigors). Vital sign cutoffs were determined using age-specific ranges defined by Goldstein, et al (54), and chemotherapy regimen intensity was categorized as least (level 1), moderate (level 2), very (level 3), or most (level 4) intensive based on the Intensity of Treatment Rating Scale (ITR-3.0) as previously defined (55). Maximum temperature (Tmax) was determined by the Tmax reported by the patient caregiver prior to arrival, or the Tmax documented within the first two hours of ED arrival, whichever value was higher.

The CDE outcome was met if the patient experienced ≥1 CDE within 48 hours of ED arrival. A CDE was characterized as a significant change in clinical status, as previously defined (56) by the following qualifying events: transfer from a pediatric ward to PICU, respiratory failure (initiation of non-invasive positive pressure ventilation (NIPPV) or endotracheal intubation), administration of ≥60 ml/kg (or ≥3 L if weight ≥50 kg) of crystalloid bolus intravenous fluids (IVF) in a 24-hour period, vasopressor or inotrope initiation, altered mental status, or death (56). Bolus IVF administration was based on provider discretion. In patients with chronic mechanical ventilatory needs, respiratory failure was defined as a need for increased ventilator settings above baseline.

A separate analysis was performed evaluating the occurrence of at least one (≥1) IBI within 48 hours of ED arrival. IBI was defined as the isolation of a bacterial organism from a normally sterile body fluid (i.e., blood, urine, cerebrospinal fluid, pleural fluid) (57), lobar pneumonia identified by chest radiograph (CXR) or computed tomography (CT) scan, intraabdominal infection, or skin/soft tissue infection (SSTI) necessitating IV antibiotics. Bacterial identification *via* blood culture was only included as an IBI if the result was not considered to be a contaminant (58) and resulted in a full antimicrobial treatment course for bacteremia.

Secondary outcomes included: hospital length of stay (LOS), PICU admission, PICU LOS, non-invasive infection within 48 hours, and 30-day mortality. Given that the clinical implications of an IBI exceed those of a non-invasive infection, analysis of non-invasive infection as a secondary outcome did not include encounters among patients who were diagnosed with an IBI within 48 hours. Thirty-day mortality was included as a descriptive outcome only due to the low incidence in the cohort.

### Statistical analysis

Analysis was performed using R version 4.0.2 and the significance level was set to 0.05. Variables were summarized using median (interquartile range, IQR) or frequency (percentage) for each encounter. Receiver operating characteristic (ROC) curves were reported for each outcome (CDE and IBI) with with lactate level as the sole predictor as the sole predictor. PHO patients with multiple fever encounters have the possibility of introducing correlation with their specific patient characteristics, therefore generalized estimating equations (GEE) with a logit link were used to model risk factors for CDE and IBI within 48 hours using an exchangeable correlation structure to account for correlation among patients with multiple encounters. Univariate models were fit for each risk factor of interest with lactate level included both as a continuous and categorical variable, utilizing the frequently reported cut offs in the literature of 0-2 mmol/L, 2-4 mmol/L, and ≥4 mmol/L (29, 34, 43, 59–64). Variables in the univariate model were considered for selection in a multivariable model for each outcome based on significance and clinical relevance. Lactate level (continuous) was forced in, and the final set of predictors was selected based on the lowest Quasi-likelihood under Independence Model Criterion (QIC), a metric that assesses the degree to which data fits the GEE model and can be used for covariate selection (65). The QIC for the final multivariable models with and without lactate were established. Lower QIC values indicate better model fit, and a difference of 2-4 units is considered meaningfully different.

Lactate was tested for association with secondary outcomes. GEE was used to model PICU admission and non-invasive infection (binomial) and hospital LOS, PICU LOS, and vasopressor duration (gaussian). Continuous outcomes were log transformed before modeling due to non-normality, and results were back-transformed for reporting. The R package gee was used for modeling and reproducible code can be found here: https://github.com/campbkri/lactate_paper.

## Results

### Patient characteristics

**Figure 1:**
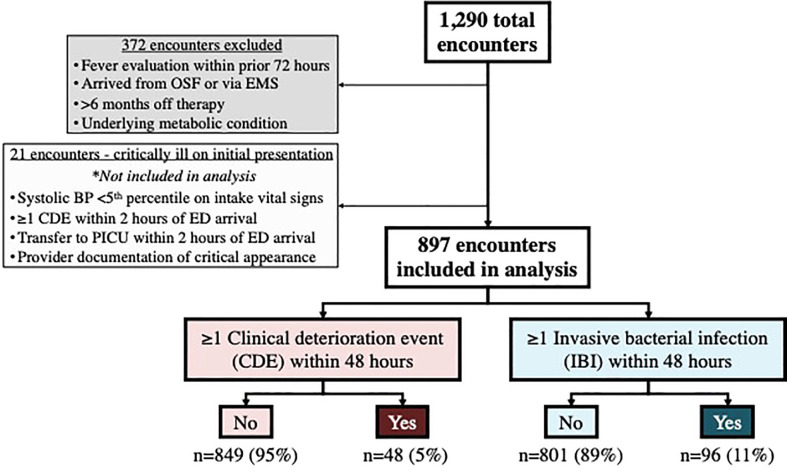
Study flowchart. OSF, Outside facility; EMS, Emergency medical services; BP, Blood pressure; CDE, Clinical deterioration event; ED, Emergency department; PICU, Pediatric intensive care unit; IBI, Invasive bacterial infection.

As outlined in the study flowchart in **Figure 1**, there were 1290 total eligible encounters, among which 372 were excluded. In an additional 21 encounters, the patient appeared critically ill at presentation; clinical data and outcomes for these 21 encounters are described separately below, but were otherwise excluded from the remainder of these analyses. Encounter data and relevant initial lab values for the remaining 897 encounters included in the analysis are listed in **Table 1**, including those with occurrence of one or more (≥1) CDE within 48 hours (n=48 encounters among 45 patients), and one or more (≥1) IBI within 48 hours (n=96 encounters among 85 patients).

**Table 1:** Patient encounter characteristics and initial laboratory values.

	All encounters (n=897)	CDE within 48 hours (n=48)	IBI within 48 hours (n=96)
Characteristic	n (%)	n (%)	n (%)
**Number of unique patients**	456	45	85
**Age in years, *median (IQR)***	6.5 (3.8, 11.7)	12.4 (7.0, 15.7)	6.9 (3.1, 12.9)
**Sex**			
Female	366 (41%)	19 (40%)	45 (47%)
Male	531 (59%)	29 (60%)	51 (53%)
**Underlying Diagnosis**			
Acute lymphoblastic leukemia	421 (47%)	22 (46%)	43 (45%)
Acute myeloid leukemia	13 (1%)	1 (2%)	2 (2%)
Lymphoma	65 (7%)	4 (8%)	7 (7%)
Solid Tumor	277 (31%)	17 (35%)	30 (31%)
CNS Tumor	116 (13%)	4 (8%)	13 (14%)
^*^Other	5 (0.6%)	0 (0%)	1 (1%)
**Phase of therapy**			
^†^On therapy	885 (99%)	48 (100%)	98 (96%)
New diagnosis during ED encounter	5 (0.6%)	0 (0%)	0 (0%)
Off therapy within **<**6 months	7 (0.8%)	0 (0%)	4 (4%)
**HSCT within past 6 months, *yes***	52 (6%)	3 (6%)	12 (12%)
Allogeneic	18 (2%)	0 (0%)	8 (8%)
Autologous	34 (4%)	3 (6%)	4 (4%)
**^††^Chemotherapy intensity**			
Most (level 4)	94 (11%)	9 (18%)	17 (18%)
Very (level 3)	529 (59%)	25 (52%)	56 (58%)
Least/moderate (levels 1&2)	267 (30%)	14 (29%)	22 (23%)
Unknown/other	7 (0.8%)	0 (0%)	1 (1%)
**Venous catheter type**			
Implanted port	769 (86%)	38 (79%)	67 (70%)
External tunneled catheter	102 (11%)	8 (17%)	24 (25%)
PICC line	10 (1%)	1 (2%)	4 (4%)
Peripheral IV	15 (2%)	1 (2%)	1 (1%)
**^¶^Initial ED laboratory values**			
Lactate in mmol/L, *median (IQR)*	1.4 (1.0, 2.0)	2.0 (1.4, 3.0)	1.7 (1.2, 2.3)
Lactate **<**2 mmol/L, *categorical* (n, %)	665 (74%)	21 (44%)	61 (64%)
Lactate 2-4 mmol/L, *categorical* (n, %)	200 (22%)	20 (42%)	35 (36%)
Lactate ≥4 mmol/L, *categorical* (n, %)	32 (4%)	7 (15%)	10 (10%)
Absolute monocyte count (x10^3^/μL)	0.22 (0.03, 0.54)	0.06 (0.01, 0.30)	0.04 (0.01, 0.27)
Absolute lymphocyte count (x10^3^/μL)	0.38 (0.16, 0.88)	0.27 (0.09, 0.75)	0.21 (0.08, 0.65)
Absolute neutrophil count (x10^3^/μL)	0.76 (0.04, 3.57)	0.13 (0.01, 1.46)	0.06 (0.01, 2.48)
^§^Neutropenic, yes (n, %)	414 (46%)	29 (60%)	63 (66%)

CDE, Clinical deterioration event IBI, Invasive bacterial infection; IQR, Interquartile range; CNS, Central nervous system; PICC, Peripherally inserted central catheter; HSCT, Hematopoietic stem cell transplantation; ED, Emergency department.

^*^Other: Aplastic anemia (n=3), antiphospholipid syndrome (n=1), β-thalassemia (n=1).

**^†^** Includes patients receiving chemotherapy or within 6 months of HSCT.

^††^Based on Intensity of Treatment Rating criteria (Kazak, et al. Pediatric Blood & Cancer, 2012).

^¶^Initial values within two hours of ED arrival (lactate in mmol/L and white blood cell counts) are reported as median (IQR). Categorical lactate levels and presence of neutropenia are reported as n (%).

^§^Neutropenia defined as absolute neutrophil count **<**0.5 x10^3^/μL.

The median age for the overall cohort was 6.5 years (IQR: 3.8-11.7). Leukemia/lymphoma accounted for over half of underlying patient diagnoses (55%), followed by solid tumors (31%), CNS tumors (13%), and non-malignant hematologic disorders (0.6%). Similar proportions of each underlying patient diagnosis were noted among encounters that met the CDE and IBI outcomes. Almost every encounter (n=885, 99%) occurred among patients who were actively undergoing therapy, including those who had undergone HSCT (n=52) within the past six months. A small subset of patients had recently completed therapy (n=7), or were newly diagnosed with a malignancy while admitted to the ED (n=5). Chemotherapy regimen intensity varied among the cohort and regimens received characterized as very (level 3) intensive were seen most frequently (n=529, 59%). Implanted ports were the most common type of central venous access, whereas external tunneled catheters, peripherally inserted central catheter (PICC) lines, and peripheral IV’s were less common. The median initial lactate level was 1.4 mmol/L (IQR 1.0-2.0) among the overall cohort, 2.0 mmol/L (IQR 1.4-3.0) among those who had ≥1 CDE, and 1.7 mol/L (IQR 1.2-2.3) among those diagnosed with ≥1 IBI. White blood cell (WBC) counts were assessed within two hours for nearly all encounters (the absolute monocyte count was not reported for one encounter) and nearly half of the febrile encounters occurred in neutropenic patients (n=414, 46%). Unlike WBC counts and venous lactate, c-reactive protein (CRP) and procalcitonin (PCT) were infrequently obtained within two hours (CRP: 3%, PCT: 1%).

### Association of lactate level with clinical deterioration events within 48 hours

**Figure 2:**
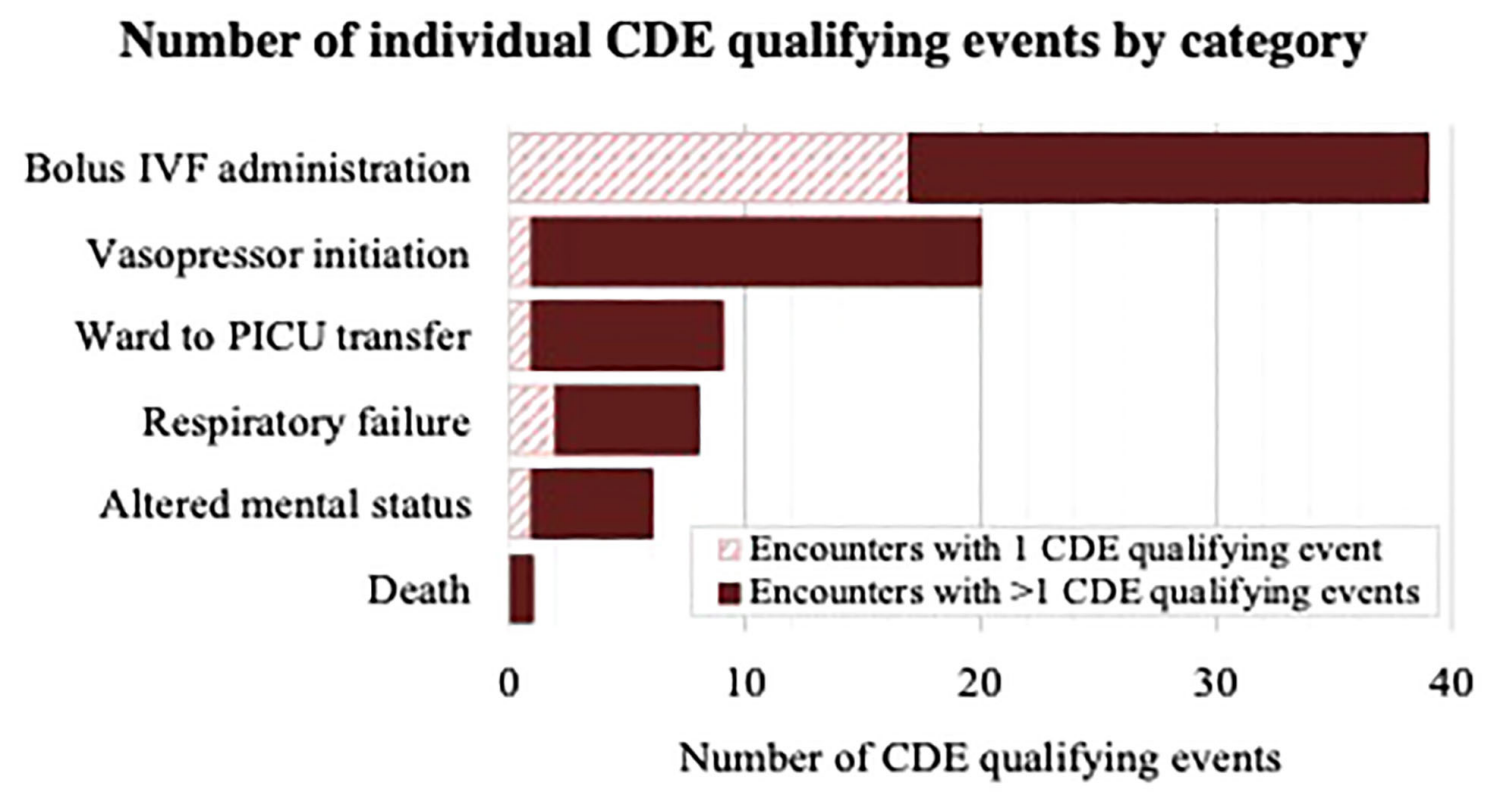
Diagram demonstrating number of CDE qualifying events per category (bolus IVF administration, vasopressor initiation, ward to PICU transfer, respiratory failure, altered mental status, death) among encounters with one CDE qualifying event and encounters with multiple CDE qualifying events. IVF, Intravenous fluid; PICU, Pediatric intensive care unit.

At least one (≥1) CDE occurred within 48 hours in 48 of the 897 included encounters (5%). In 22 of these, one isolated CDE qualifying event occurred, whereas multiple CDE qualifying events occurred in the remaining 26, together accounting for 83 total individual CDE qualifying events among the entire cohort, shown in **Figure 2**. The most common categories of CDE qualifying events were bolus IVF administration (n=39, 47% of all CDEs) and initiation of vasopressors (n=20, 24% of all CDEs), whereas ward to PICU transfer (n=9, 11% of all CDEs), respiratory failure (n=8, 10% of all CDEs), altered mental status (n=6, 7% of all CDEs), and death (n=1, 1% of all CDEs) occurred less frequently.

**Figure 3:**
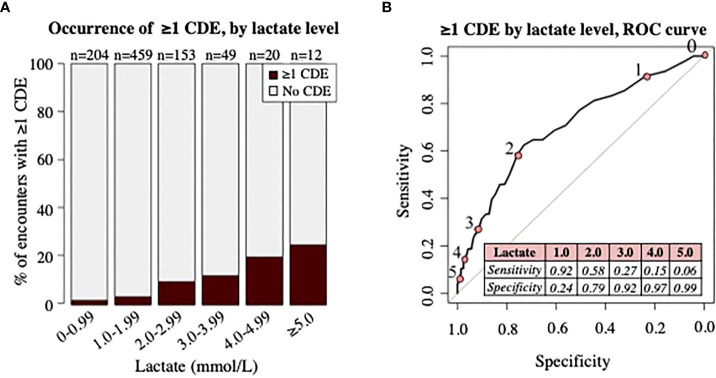
Analysis of clinical deterioration events (CDE) by lactate level. **(A)** Proportion of patient encounters with occurrence of ≥1 CDE by lactate level in increments of 1mmol/L. Numbers (n) on top of bars signify the total number of patient encounters with initial lactate level in specified range. **(B)** ROC curve demonstrating association of lactate level with occurrence of ≥1 CDE. Numbers 0-5 along ROC curve represent ROC curve points for lactate level cutoffs (pink circles) in mmol/L, shown in the table. Area under the curve = 0.704.

Comparing the distribution of initial lactate levels and CDE occurrence revealed an increased proportion of patient encounters with ≥1 CDE with incremental increases in initial lactate level (**Figure 3A**). At least one CDE was seen in four of 204 encounters (2%) with lactate <1.0 mmol/L, 16 of 459 encounters (4%) with lactate 1-1.99 mmol/L, 15 of 153 encounters (10%) with lactate 2-2.99 mmol/L, 6 of 49 encounters (12%) with lactate 3-3.99 mmol/L, 4 of 20 encounters (20%) with lactate 4-4.99 mmol/L, and 3 of 12 encounters (25%) with lactate ≥5 mmol/L. The ROC curve (AUC 0.704) shown in **Figure 3B** demonstrates the sensitivity and specificity of individual lactate level cutoffs for predicting the occurrence of ≥1 CDE within 48 hours.

### Univariate analysis results, occurrence of ≥1 CDE by risk factor

Results of the univariate analysis testing the association of lactate level and covariates with the occurrence of ≥1 CDE within 48 hours are shown in **Supplemental Table 1**. The odds of clinical deterioration increased by 77% with each unit increase in lactate level (p<0.001). Evaluation of lactate level using previously reported cutoffs revealed increased odds of ≥1 CDE with moderate lactate elevation 2-4 mmol/L (OR 3.74, 95%CI: 2.00-7.01, p<0.001), and even higher odds with lactate levels ≥4 mmol/L (OR 8.82, 95% CI: 3.51-22.20, p<0.001), when compared to those with lactate < 2 mmol/L. Older age (p<0.001), vital sign abnormalities including hypotension (p<0.001) and tachycardia (p<0.001) within the first two hours of ED arrival, chills or rigors (p<0.05), and neutropenia (p<0.05) were also associated with the occurrence of ≥1 CDE in the unadjusted analysis, whereas underlying diagnosis, chemotherapy regimen intensity, recent HSCT, type of venous access, Tmax, presence of tachypnea within two hours, and WBC counts were not.

### Multivariable analysis results, occurrence of ≥1 CDE by risk factor

The optimal set of predictors for inclusion in the multivariable model based on QIC were lactate level (continuous), age, chemotherapy regimen intensity, and presence of hypotension and tachycardia within two hours. Given the clinical relevance of neutropenia at the time of fever in PHO patients, a sensitivity analysis was performed with neutropenia added into the same model, which showed a non-significant association of neutropenia with having ≥1 CDE (p=0.17) and a higher QIC, thus neutropenia was not forced into the model. The results of the multivariable analysis are shown in **Table 2**. After adjusting for age, chemotherapy regimen intensity, and the presence of hypotension and tachycardia within two hours of ED arrival, increased lactate level was significantly associated with occurrence of ≥1 CDE (OR 1.82, 95% CI: 1.43-2.32, p<0.001). After controlling for covariates, odds of ≥1 CDE were increased with older age (OR: 1.13, 95% CI: 1.07-1.19, p<0.001). Outcomes also differed significantly among chemotherapy regimen intensity groups (p<0.05). Presence of hypotension (OR: 3.78, 95% CI: 2.64-15.99, p<0.001) and tachycardia (OR: 3.78, 95% CI: 1.61-8.84, p<0.01) within two hours of ED arrival were also significant after adjusting for confounding variables. The QIC of the multivariable model without lactate level was 325, whereas the QIC of the model with lactate level included was 305. This difference in 20 points of QIC indicates a significantly better fit of the model when lactate was included.

**Table 2:** Occurrence of ≥1 clinical deterioration event (CDE) within 48 hours, adjusted odds by risk factor (results of multivariable analysis).

Risk Factor	Reference	Odds Ratio	95% CI	p value
**Lactate (mmol/L), *continuous***	–	1.82	1.43, 2.32	<0.001
**Age in years**	–	1.13	1.07, 1.19	<0.001
**^†^Chemotherapy intensity**	Most (level 4)			0.03
Very (level 3)		0.30	0.11, 0.78	0.01
Least/moderate (levels 1&2)		0.56	0.21, 1.51	0.25
*ED clinical status				
Hypotension	No	6.49	2.64, 15.99	<0.001
Tachycardia	No	3.78	1.61, 8.84	<0.01
**QIC of model *without* lactate: 325, QIC *with* lactate: 305**				

QIC, Quasilikelihood under the Independence model Criterion; ED, Emergency department.

*Based on Intensity of Treatment Rating criteria (Kazak, et al. Pediatric Blood & Cancer, 2012).

^†^Hypotension and tachycardia refer to presence of age-based vital sign abnormalities within two hours of ED arrival.

### Association of lactate level with incidence of invasive bacterial infection (IBI) within 48 hours

Within 48 hours of ED arrival, at least one (≥1) IBI was diagnosed in 96 of 897 encounters (11%), including 16 encounters in which the patient was diagnosed with a non-invasive infection in addition to IBI within 48 hours. Frequency of IBI by source of infection and corresponding median lactate levels are outlined in **Supplemental Table 2**. Bacterial bloodstream infection (BSI) was the most common source of IBI, seen in 58 encounters (6%). Other sources of IBI were less common, including pneumonia (n=18, 2%), genitourinary (GU, n=10, 1%), SSTI (n=10, 1%), and intraabdominal (n=6, 0.7%). Median initial lactate levels were similar across encounters with ≥1 IBI regardless of infection source with exception of a relatively higher median initial lactate (2.7 mmol/L, IQR 1.3-4.2) in those with intraabdominal infection, although this discrepancy may be due the infrequency of each IBI type rather than true variation.

**Figure 4:**
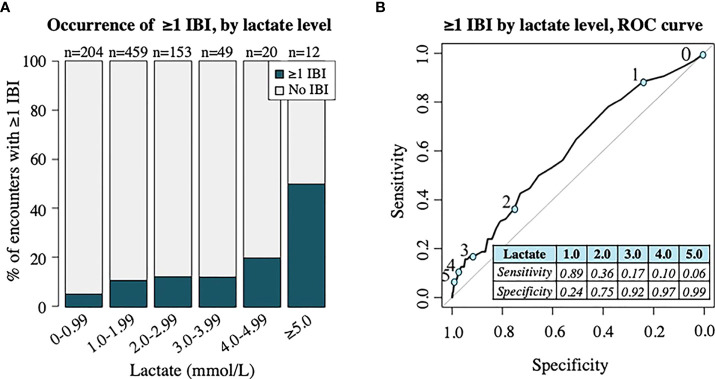
Analysis of invasive bacterial infection (IBI) by lactate level. **(A)** Proportion of patient encounters with occurrence of ≥1 IBI by lactate level in increments of 1 mmol/L. Numbers (n) on top of bars signify the total number of patient encounters with initial lactate level in specified range. **(B)** ROC curve demonstrating association of lactate level with occurrence of one ≥1 IBI. Numbers 0-5 along ROC curve represent ROC curve points (blue circles) for lactate level cutoffs in mmol/L, shown in table. Area under the curve = 0.608.

The distribution of initial lactate levels with occurrence of ≥1 IBI demonstrated in **Figure 4A** revealed an increased proportion of patients diagnosed with ≥1 IBI within 48 hours as lactate level incrementally increased, although the most notable difference occurred once lactate levels reached 4 mmol/L and above. An ROC curve (AUC: 0.608) including the sensitivity and specificity of individual lactate level cutoffs for predicting the occurrence of ≥1 IBI within 48 hours is shown in **Figure 4B**.

### Univariate analysis results, occurrence of ≥1 IBI by risk factor

Results of the univariate analysis demonstrating the association of lactate level and covariates with the occurrence of ≥1 IBI within 48 hours are shown in **Supplemental Table 3**. For each unit increase in lactate level, the odds of being diagnosed with ≥1 IBI within 48 hours increased by 40% (p<0.001). When compared to patients with lactate levels <2 mmol/l, categorical evaluation of lactate level demonstrated increased odds of ≥1 IBI with lactate level ≥4 mmol/L (OR: 4.34, 95% CI: 1.91-9.86, p<0.001), but no significant difference in patients with moderately elevated lactate 2-4 mmol/L (p=0.20). Compared to patients with implanted ports, those with external tunneled catheters (OR: 3.21, 95% CI: 1.83-5.65, p<0.001) and peripherally inserted central catheter (PICC) lines (OR: 7.00, 95% CI: 1.82-26.94, p<0.01) were associated with increased IBI. The presence of chills or rigors was associated with increased odds of ≥1 IBI (OR: 2.35, 95% CI: 1.15-4.81, p<0.05).

There was no association between occurrence of ≥1 IBI and initial WBC counts when analyzed continuously, including ANC, absolute monocyte count (AMC), and absolute lymphocyte count (ALC); however, neutropenic patients had 2.5-times higher odds of ≥1 IBI when compared to non-neutropenic patients (p<0.001). No association was observed between age or underlying diagnosis and the occurrence of ≥1 IBI. Outcomes did not significantly differ among chemotherapy regimens (p=0.06), however compared to those in the most (level 4) intensive category, the diagnosis of IBI was less common as chemotherapy regimen intensity decreased. Despite the increased incidence of IBI seen in patients with tachycardia and hypotension, the lack of a significant association between these vital sign abnormalities and IBI was an unexpected finding.

### Multivariable analysis results, occurrence of ≥1 IBI by risk factor

Covariates included in the multivariable analysis based on QIC were lactate level (continuous), type of venous access, presence of chills or rigors, and presence of neutropenia as shown in **Table 3**. The QIC of the model with and without lactate included was 563 and 579, respectively. A difference of 16 points of QIC indicates the model with lactate fits the data significantly better than the model without lactate.

**Table 3:** Occurrence of ≥1 invasive bacterial infection (IBI) within 48 hours, adjusted odds by risk factor (results of multivariable analysis).

Risk Factor	Reference	Odds Ratio	95% CI	p value
**Lactate (mmol/L), *continuous***	–	1.49	1.23, 1.79	< 0.001
**Type of venous access**	Implanted port			< 0.001
External tunneled catheter		4.28	2.32, 7.87	< 0.001
PICC line		5.56	1.29, 23.90	0.02
Peripheral IV		0.77	0.10, 6.19	0.81
**Chills or rigors**	No	2.23	1.04, 4.80	< 0.001
*Neutropenic, yes	No	2.54	1.60, 4.03	< 0.001
**QIC of model *without* lactate: 579, QIC *with* lactate: 563**				

PICC, Peripherally inserted central catheter; QIC, Quasilikelihood under the Independence model Criterion.

^*^Neutropenia defined as absolute neutrophil count **<**0.5 x10^3^/μL.

Elevated lactate level (OR: 1.49, 95% CI: 1.23-1.79, p<0.001), neutropenia (OR: 2.54, 95% CI: 1.60-4.03, p<0.001), and presence of chills or rigors (OR: 2.23, 95% CI: 1.04-4.80, p<0.001) were all associated with increased odds of ≥1 IBI after adjusting for covariates. Additionally, patients with external tunneled catheters (OR 4.28, 95% CI: 2.23-7.87, p<0.001) and PICC lines (OR 5.56, 95% CI: 1.29-23.90, p<0.05) had increased odds of ≥1 IBI compared to those with implanted ports in the adjusted analysis, whereas those with peripheral IVs did not (p=0.81).

### Association of initial lactate level with secondary outcomes

In addition to CDE and IBI, increased lactate level was associated with secondary outcomes pertaining to illness severity as shown in **Supplemental Table 4**. Among all encounters, the median hospital LOS was 3.8 days (IQR 2.5-6.8) for patients admitted from the ED. PICU admission was required in 55 encounters (6%), and the median PICU LOS was 1.4 days (IQR 0.9-2.7). With each 1 mmol/L increase in lactate level, there was an associated 15% increase in hospital LOS, 68% higher odds of being admitted to the PICU during hospitalization, and 21% increase in PICU LOS (all p values <0.05).

One or more non-invasive infection(s) were diagnosed within 48 hours in 168 encounters (19%), excluding the 16 in which patients who were diagnosed with both IBI and non-invasive infection(s). Sources of non-invasive infection and median lactate levels are shown in **Supplemental Table 2**. Viral URI was the most common source of non-invasive infection (n=125, 14%) in this group. Lactate level did not significantly differ among patients who were diagnosed with non-invasive infection(s) only and those who were not diagnosed with any infection within 48 hours (p=0.09). Thirty-day mortality was low for the cohort. Six patients died within 30 days of ED presentation (<1%), two from infection-related causes, and four from underlying disease progression. In those six patients, the median time to death was 16.5 days (range 2-26 days) from ED arrival. The two infection-related deaths included one patient with T-cell acute lymphoblastic leukemia (ALL) undergoing delayed intensification therapy who died 10 days after ED presentation due to septic shock with multi-organ failure from disseminated fungal infection and a patient with progressive metastatic atypical rhabdoid tumor (ATRT) found to have to have widely disseminated fungal infection during hospitalization. Due to poor prognosis, all antifungals were discontinued, and he was discharged on hospice care, and ultimately died 19 days after ED presentation.

### Patients who appeared critically ill upon ED arrival

Here we describe the 21 encounters among 20 patients (median age 13.3 years, IQR 3.7-16.0) who were critically ill appearing upon initial ED presentation, summarized in **Supplemental Table 5**. These patients were not included in the remainder of the analysis and expectedly had worse outcomes than the remainder of the cohort. Underlying diagnoses varied, including acute lymphoblastic leukemia (ALL, n=6), acute myeloid leukemia (AML, n=2 encounters for one patient), lymphoma (n=12), solid tumors (n=5), CNS tumors (n=2), and non-malignant hematologic disorders (n=2). Three patients had undergone HSCT within six months prior to presentation. The median initial lactate level in this ill-appearing cohort was 3.2 mmol/L (IQR 1.7-5.1), the median ANC was 0.54 x 10^3^/μL (IQR 0.01-3.62), and almost half of the patients were neutropenic at presentation (10 of 21 encounters, 48%). In all but one encounter (95%) the patient experienced ≥1 CDE within 48 hours, and the majority (n=14) experienced multiple CDE qualifying events rather than a single CDE qualifying event (n=6). At least one IBI was diagnosed within 48 hours in two-thirds of the encounters (n=14). The most common type of IBI was BSI (n=14) whereas focal infections (pneumonia: n=3, intraabdominal: n=1, GU: n=1) were less common. Five patients (25%) died within 30 days of ED presentation (median duration from ED arrival to death: 8 days, range 0-26 days). Four deaths were infection-related, and one patient died from underlying disease progression.

## Discussion

PHO patients are at high risk for life-threatening infectious complications and progression to sepsis/septic shock. Prompt detection and resuscitation is critical for improving outcomes, which can be challenging as PHO patients often lack typical signs of illness and may present with fever as the sole sign of occult infection and impending deterioration (66). Thus, understanding the implications of objective laboratory markers in PHO patients specifically is essential for the development of superior risk stratification strategies in this group.

Serum lactate is well-established prognostic indicator for general adult and pediatric populations (27–32), and the importance of lactate as a reliable laboratory marker is emphasized by its incorporation in the most recent adult sepsis-3 criteria (67). Results of general pediatric studies indicate an association between elevated lactate and serious bacterial infection (SBI), organ dysfunction, prolonged hospitalization, and mortality (28, 34, 43, 68). Notably, the benefit of lactate measurement has been shown to be independent of hemodynamic variables and organ dysfunction (29, 33, 34).

Although serum lactate has been evaluated as a prognostic indicatior to some extent in adult oncology patients, data is limited and contradictory (30, 69, 70). There is minimal data regarding interpretability of lactate levels in PHO patients who have substantial differences compared to their adult counterparts in terms of underlying malignancies, comorbidities, metabolism/developmental stages, infectious considerations, and increased treatment regimen intensities (10, 71). We report the association of initial lactate level with poor outcomes in the largest study assessing this laboratory marker in PHO patients to date. Although two existing studies in PHO patients similarly describe an association with lactate levels and illness severity (47, 48), these studies are considerably limited due to small sample size.

Importantly, we demonstrate a similarly reported association between serum lactate levels and increased illness severity in other patient populations, suggesting these values are not routinely elevated or uninterpretable in PHO patients despite the known impact that malignancy, chemotherapy, and organ dysfunction have on lactate metabolism. Given the strength of these data, they provide the basis for further investigation of serum lactate as a tool that can be incorporated into risk stratification models to optimize detection of patients at high risk for deterioration.

Our results demonstrate an association between lactate level and clinical deterioration in PHO patients when analyzed both as a continuous variable and a categorical variable using cutoffs that have been most frequently reported in the literature. While interpretation of this type of laboratory marker using distinct cut points such as ≥2 mmol/L or ≥4 mmol/L rather than as a continuous value may be more practical in the clinical setting, studies in other populations have yielded similar results to our findings: severe outcomes increase linearly with increases in lactate without a clear clinical inflection point (72). Prospective studies validating the utility of this marker as a stratification tool may require different cut points based on the goal of ruling in or ruling out patients at risk for deterioration.

In efforts to capture patients with undifferentiated illness severity at initial evaluation, patients who were critically ill-appearing at presentation were not included in the analysis, and this group predictably had worse outcomes. It is important to note that provider determination of illness severity should remain the primary basis for escalation of care, regardless of what is dictated by any risk prediction model or laboratory result. While the median lactate level was notably higher in this group, this result adds minimal clinical benefit in the context of an ill-appearing patient but does support the concept of elevated lactate as a physiologic response to critical illness in PHO patients.

Among all patients with CDEs, the frequency of IV fluid resuscitation and vasopressor initiation we report are concordant with sepsis as a well-established cause of clinical deterioration in PHO patients (6, 73–75). Moreover, the exclusion of patients who were critically ill at the time of febrile presentation supports the notion that PHO patients may not initially demonstrate classic signs of severe illness with fever but remain at risk for rapid deterioration from sepsis in the subsequent hours. Compared to prior studies which cite respiratory failure as an equal or more frequent contributor to clinical deterioration in this population (7, 73, 76, 77), it only represented 10% of all CDE qualifying events in this study. This may be attributed to multiple factors including the ED setting of this study resulting in exclusion of patients at risk for different complications (e.g., AML and immediate post-HSCT patients), exclusion of patients who were immediately ill-appearing from the overall analysis, and variable definitions of respiratory failure utilized in the literature.

In addition to increased lactate level, older age, highly intensive chemotherapy regimens, and the presence of tachycardia and/or hypotension within two hours of ED arrival were all associated with increased CDE within 48 hours in an adjusted model. The substantial improvement of the multivariable model based on the QIC seen when lactate was included with the remaining risk factors suggests that lactate level can provide additional benefit to other established predictors of illness severity. Hypotension as a predictive variable is difficult to interpret given its intricate link to two potential CDEs (IVF resuscitation and vasopressor initiation), thus the strong association with the primary outcome (≥1 CDE) was expected. Importantly, increased lactate level maintained a significant association with CDE after controlling for the presence of hypotension, supporting the notion that hypotension may be a late finding in pediatric sepsis (78), and lactate elevation can denote inadequate perfusion despite normal blood pressure values (33, 34).

Unlike many studies evaluating risk factors for deterioration in the PHO patient population, we elected to include non-neutropenic patients. In addition to identifying which patients need urgent intervention, there is also significant interest in risk models that enable decreased intervention for patients at lower risk, which largely includes the non-neutropenic population. The incidence of CDE was significantly higher in neutropenic patients compared to non-neutropenic patients. Despite this, neutropenia was not determined to be a necessary variable in the multivariable model of risk factors for CDE. Not only did the best-fit of the model decrease when neutropenia was incorporated, but the presence of neutropenia was no longer significant when evaluated in the context of other relevant covariates. This suggests that although the presence and duration of neutropenia is associated with risk of severe infection, there is likely a role for incorporation of additional clinical and laboratory factors to improve risk prediction for clinical decompensation specifically.

While often linked with clinical deterioration in PHO patients, IBI was included in this study as a separate outcome to test the association of lactate level with serious infection, regardless of clinical illness severity. This has implications for potential incorporation of serum lactate into future prediction models targeted towards decision-making about antimicrobial administration. Our overall incidence of IBI was lower than reported in other studies of PHO patients (79–83), however this was expected given our inclusion of non-neutropenic patients. The majority of studies reporting infectious outcomes in PHO are limited to neutropenic patients as neutropenia is an established risk factor for bacterial infection, which is in accordance with our study results.

As an isolated risk factor, increased lactate level was significantly associated with increased odds of IBI, although distinct differences in IBI rates were not appreciated unless lactate was substantially elevated to levels ≥4 mmol/L. A possible explanation for this is that lactate may not be as tightly associated with IBI, compared to CDE, due to lactate being a marker of tissue hypoperfusion and organ dysfunction regardless of etiology. In other words, lactate is more suitable for detecting the negative downstream effects of severe infection, rather than infection itself.

Despite the trend toward increased incidence of IBI seen in patients with tachycardia and hypotension, the lack of a significant association between these vital sign abnormalities and IBI was an unexpected finding. This result may represent an insufficient immune response in the setting of bacterial infection due to immunosuppressive therapies, which further emphasizes the need for improved strategies to determine infection risk in this group. It is also possible that a difference was not appreciated as more discrete measures of the degree of tachycardia and hypotension such as z-scores were not utilized (84). In accordance with prior studies, external central catheters were associated with increased IBI (85, 86), although this may have been influenced in part by the underlying diagnoses and treatment regimens that mandate external catheters versus implanted ports. Additionally, chills or rigors reported by patients or documented by ED providers was an important risk factor for IBI, suggesting that this should be part of the routine evaluation of PHO patients presenting with fever. As described above, substantial improvement in the multivariable model of risk factor association with IBI seen with the incorporation of lactate level demonstrates that it can provide additional benefit in distinguishing which patients are at highest risk for IBI. Moreover, elevated lactate level was associated with IBI but not non-invasive infection types. This suggests a role in specifically distinguishing patients with the most clinically significant infection types. This has important implications for risk stratification strategies as there is significant interest in improving our ability to distinguish which patients require aggressive intervention with broad-spectrum antimicrobials from those who would benefit from less intensive therapy.

In accordance with other pediatric studies, we chose clinical deterioration events as a primary marker of illness severity in addition to other secondary outcomes that signify more severe illness including PICU admission and LOS, duration of hospitalization, and mortality. While mortality is the most extreme predictor of illness severity, mortality rates are relatively low in our pediatric population as seen in this study, and clinical deterioration events still hold significant implications for both short and long-term healthcare outcomes and quality of life. We demonstrated longer length of hospitalization, increased rates of PICU admission, and longer PICU LOS as lactate level increased, suggesting this cohort represents a sicker group of patients. The longer hospital LOS seen with elevated lactate level may represent a subacute difference in illness severity. Although the increase in hospital LOS may seem inconsequential, it approximates to an additional day of hospitalization above the median LOS for every 1 mmol/L increase in initial lactate level. Thirty-day infection-related mortality was exceptionally low for the overall cohort, which is in line with improvements seen in supportive care practices for critically ill patients over the last several decades. We suspect that in a larger cohort, there would be a significant difference in mortality, especially in a shorter time-period following a septic event.

The use of lactate in monitoring hemodynamic resuscitation in general populations of children with septic shock is recommended in the evidence-based consensus guidelines of the Surviving Sepsis Campaign and is already common practice in many pediatric EDs in the care of sepsis (78, 87). While this study cannot establish exactly which clinical actions should be taken based on a specific lactate level in a PHO patient, it gives support to this practice in PHO patients with suspected sepsis in the ED, in alignment with current standard of care for all children with sepsis. Further research may establish specific considerations needed to interpret lactate in PHO patients, but it is a low-cost, readily available laboratory test already strongly supported in pediatric sepsis generally, and it is likely to aid in early detection and monitoring of PHO patients, as it already does non-PHO children with sepsis.

There are limitations to this study, including the retrospective nature and unblinded analysis. While our results indicate an association between initial lactate level and increased severity of illness in febrile PHO patients, the most effective way to incorporate serum lactate levels in the clinical setting cannot be derived from these study results alone. The utility of any predictive laboratory marker cannot be established without understanding its meaning in the context of other relevant risk factors. We attempted to account for this by demonstrating that models including known risk factors for CDE and IBI were superior when lactate level was included.

This study is subject to selection bias as it was limited to a single-center tertiary care center that included only ED encounters, which may limit generalizability. Utilizing this available ED database allowed for substantial patient numbers but resulted in omission of patients who are already admitted to the inpatient unit at the time of fever. While this study included a larger number of febrile episodes compared to other studies of serum lactate in PHO patients due to inclusion of non-neutropenic patients, the number of neutropenic patients in this study (n=414) still far exceeded the number evaluated in previous studies (i.e. Suwanpakdee et al. n=99, and Pacheco-Rosas et al. n=100). Although our results indicated that serum lactate remained a significant prognostic indicator after controlling for the presence of neutropenia as a covariate in the statistical analysis, there may be more nuanced implications for this laboratory marker if analyzed specifically in neutropenic versus non-neutropenic patients. Additionally, patients who were transferred from an outside institution or *via* EMS were excluded to ensure that patients had not undergone interventions prior to arrival that may impact lactate level. This limits the potential study population to patients who live within one-hour driving distance, hereby creating a demographically restricted subject group.

Outcomes may have been influenced by performance bias because clinicians were not blinded to the lactate levels, and they may have specifically carried out interventions such as fluid administration directly in response to elevated lactate levels, which would bias towards supporting the study hypothesis. Conversely, performance bias may have led clinicians to deliver more timely, high quality care to patients with elevated lactate levels, because they were aware and concerned about this lab value. If this were the case, this would potentially disproportionately improve outcomes in the high-lactate patients. Notably, we expect that this effect would have resulted in diminished variance between groups based on lactate levels, and bias towards the null hypothesis. As described above, the link between vital sign abnormalities such as hypotension and the CDE outcome were unavoidable. We elected to include this to ensure that we considered the utility of serum lactate after accounting for typical signs and symptoms of illness severity.

In conclusion, this is the largest study to date that demonstrates the association of initial serum lactate levels with adverse clinical outcomes in PHO patients specifically, who have unique metabolic considerations in the setting of malignancy and treatment regimens. While clinical decision making cannot be made based on an isolated laboratory value, this study suggests that there may be a role for serum lactate as a tool that can be incorporated into other clinical prediction tools in this unique population. The association between serum lactate and poor outcomes in PHO patients demonstrated in this study provides a foundation for future prospective investigations into the most efficacious use of this marker for this group in the future.

## Data availability statement

The original contributions presented in the study are included in the article/**Supplementary Material**. Further inquiries can be directed to the corresponding author.

## Ethics statement

The studies involving human participants were reviewed and approved by Approved by the University of Colorado Cancer Center Protocol Review and Monitoring System and the Colorado Multiple Institutional Review Board (#21-2600). Written informed consent from the participants’ legal guardian/next of kin was not required to participate in this study in accordance with the national legislation and the institutional requirements.

## Author contributions

Contribution: LS, AE, HS, ML, KM, AF, and AL-S designed analyses and analyzed data. LS and KM performed analyses. LS, AE, HS, ML, KM, AF, and AL-S wrote the manuscript. All authors contributed to the article and approved the submitted version.

## Funding

This work was supported by the University of Colorado pediatric hematology/oncology/bone marrow transplantation fellowship training program. Salary support for contributors to this work was supported by the National Institutes of Health (5K12HD068372, ABLS), Amazon Goes Gold^®^ (LRS), Swim Across America (LRS), the Conroy Family Young Investigator Endowed Fund (LRS), and the Agency for Healthcare Research and Quality (K08 HS025696, HFS).

## Acknowledgments

This work was supported by the National Institutes of Health (5K12HD068372, ABLS), Amazon Goes Gold^®^ (LRS), Swim Across America (LRS), the Conroy Family Young Investigator Endowed Fund (LRS), and the Agency for Healthcare Research and Quality (K08 HS025696, HFS).

## Conflict of interest

The authors declare that the research was conducted in the absence of any commercial or financial relationships that could be construed as a potential conflict of interest.

## Publisher’s note

All claims expressed in this article are solely those of the authors and do not necessarily represent those of their affiliated organizations, or those of the publisher, the editors and the reviewers. Any product that may be evaluated in this article, or claim that may be made by its manufacturer, is not guaranteed or endorsed by the publisher.
