# Supplementary material for: Serum lactate is associated with increased illness severity in immunocompromised pediatric hematology oncology patients presenting to the emergency department with fever
Source: Front Oncol. 2022 Oct 6;12:990279. doi: 10.3389/fonc.2022.990279 (PMC9583361; doi:10.3389/fonc.2022.990279)
Supplement: Supplementary file 1 [file DataSheet_1.docx]

Supplementary Material

| Supplemental Table 1: Occurrence of ≥1 clinical deterioration event (CDE) within 48 hours, unadjusted odds by risk factor (results of univariate analysis) | | | | |
| --- | --- | --- | --- | --- |
| Risk factor | **Reference** | **Odds Ratio** | **95% CI** | **p value** |
| *Initial lactate level |  |  |  |  |
| Lactate (mmol/L), *continuous* | - | 1.77 | 1.48, 2.12 | <0.001 |
| Lactate 2-4 mmol/L, *categorical* | Lactate <2 mmol/L | 3.74 | 2.0, 7.01 | <0.001 |
| Lactate ≥4 mmol/L, *categorical* | Lactate <2 mmol/L | 8.82 | 3.51, 22.2 | <0.001 |
| Age in years | - | 1.13 | 1.07, 1.18 | <0.001 |
| Underlying Diagnosis |  |  |  |  |
| Leukemia | All others | 1.00 | 0.54, 1.82 | 0.99 |
| Lymphoma | All others | 1.13 | 0.41, 3.13 | 0.81 |
| Solid Tumor | All others | 1.24 | 0.65, 2.37 | 0.52 |
| CNS Tumor | All others | 0.6 | 0.21, 1.74 | 0.35 |
| ^†^Chemotherapy intensity | Most (level 4) |  |  | 0.23 |
| Very (level 3) |  | 0.48 | 0.21, 1.21 |  |
| Least/moderate (levels 1&2) |  | 0.54 | 0.23, 1.31 |  |
| HSCT within prior 6 months | No | 1.07 | 0.33, 3.47 | 0.91 |
| Type of venous access | Implanted port |  |  | 0.71 |
| External tunneled catheter |  | 1.55 | 0.67, 3.57 |  |
| PICC line |  | 2.02 | 0.23, 17.89 |  |
| Peripheral IV |  | 1.24 | 0.14, 11.43 |  |
| ^††^ED clinical status |  |  |  |  |
| Maximum temperature, *continuous* | - | 1.02 | 0.97, 1.06 | 0.44 |
| Hypotension | No | 11.59 | 5.29, 25.39 | <0.001 |
| Tachycardia | No | 4.15 | 1.87, 9.20 | <0.001 |
| Tachypnea | No | 1.64 | 0.85, 3.16 | 0.14 |
| Chills or rigors | No | 2.70 | 1.11, 6.58 | 0.03 |
| URI symptoms | No | 0.83 | 0.44, 1.56 | 0.56 |
| *Initial WBC counts |  |  |  |  |
| Absolute monocyte count, *continuous* | - | 0.44 | 0.16, 1.20 | 0.11 |
| Absolute lymphocyte count, *continuous* | - | 0.73 | 0.50, 1.08 | 0.12 |
| Absolute neutrophil count, *continuous* | - | 0.94 | 0.84, 1.06 | 0.33 |
| ^¶^Neutropenic, yes | No | 1.82 | 1.003, 3.31 | 0.049 |
| Abbreviations: CNS: Central nervous system; HSCT: Hematopoietic stem cell transplantation; PICC: Peripherally inserted central catheter; ED: Emergency department; URI: Upper respiratory infection; WBC: White blood cell  *All laboratory values obtained within first two hours of ED arrival  ^†^Based on Intensity of Treatment Rating criteria (Kazak, et al. *Pediatric Blood & Cancer,* 2012)  ^††^Hypotension, tachycardia, and tachypnea refer to age-based vital sign abnormalities within two hours of ED arrival  ^¶^Neutropenia defined as absolute neutrophil count <0.5 x10^3^/μL | | | | |

| Supplemental Table 2: Sources of infection and lactate levels for patient encounters with invasive bacterial infection (IBI) or non-invasive infection diagnosed within 48 hours | | |
| --- | --- | --- |
| Invasive Bacterial Infection | | |
| *Source of infection | **n (% of all encounters)** | **Lactate in mmol/L**  *median (IQR)* |
| ≥1 IBI identified | 96 (11%) | 1.7 (1.4, 2.3) |
| Bloodstream infection (BSI) | 58 (6%) | 1.8 (1.2, 2.5) |
| Gram positive | 25 (3%) | 1.7 (1.2, 2.3) |
| Gram negative | 26 (3%) | 1.8 (1.1, 2.5) |
| ^†^Polymicrobial | 7 (0.8%) | 1.8 (1.6, 3.6) |
| Lobar pneumonia | 18 (2%) | 1.5 (1.2, 2.2) |
| Genitourinary | 10 (1%) | 1.6 (1.3, 2.1) |
| SSTI | 10 (1%) | 1.4 (0.7, 1.7) |
| Intraabdominal | 6 (0.7%) | 2.7 (1.3, 4.2) |
| ^††^Non-Invasive Infection Only | | |
| ≥1 Non-invasive infection identified | 168 (19%) | 1.3 (0.9, 2.0) |
| Viral URI | 125 (14%) | 1.4 (1.0, 2.0) |
| Gastrointestinal | 31 (3%) | 1.3 (1.0, 1.8) |
| *+Clostridium difficile* | 26 (3%) |  |
| HEENT | 8 (0.8%) | 1.0 (0.8, 1.4) |
| ^¶^Other | 3 (0.3%) | 0.8 (0.8, 1.4) |
| Abbreviations: SSTI: Skin and soft tissue infection; URI: Upper respiratory infection; HEENT: Head, eyes, ears, nose, and throat  *Patients may have had >1 source of IBI or non-invasive infection per encounter  ^†^Polymicrobial BSI: *K. pneumoniae + E. Coli; Methicillin-resistant Staphylococcus aureus (MRSA) + S. mitis; E. cloacae + K.oxytoca + S. maltophila; C. krusei + S. epidermidis; C. sojae + E. Coli; P. aeruginosa + S. viridans; E. cloacae + S. hominis*  ^††^Does not include non-invasive infection data for encounters with IBI *and* non-invasive infection within 48 hours  ^¶^Other: BK viuria, oral lesions due to varicella zoster virus, fungal dermal lesions | | |

| Supplemental Table 3: Occurrence of ≥1 invasive bacterial infection (IBI) within 48 hours, unadjusted odds by risk factor (results of univariate analysis) | | | | |
| --- | --- | --- | --- | --- |
| Risk Factor | **Reference** | **Odds Ratio** | **95% CI** | **p value** |
| *Initial lactate level |  |  |  |  |
| Lactate (mmol/L), *continuous* | - | 1.40 | 1.16, 1.69 | <0.001 |
| Lactate 2-4 mmol/L, *categorical* | Lactate <2 mmol/L | 1.40 | 0.84, 2.33 | 0.20 |
| Lactate ≥4 mmol/L, *categorical* | Lactate <2 mmol/L | 4.34 | 1.91, 9.86 | <0.001 |
| Age in years | - | 1.00 | 0.96, 1.05 | 0.85 |
| Underlying Diagnosis |  |  |  |  |
| Leukemia | All others | 0.95 | 0.61, 1.47 | 0.82 |
| Lymphoma | All others | 0.97 | 0.44, 2.15 | 0.94 |
| Solid tumor | All others | 1.01 | 0.62, 1.64 | 0.98 |
| CNS tumor | All others | 1.07 | 0.56, 2.05 | 0.84 |
| ^†^Chemotherapy intensity | Most (level 4) |  |  | 0.06 |
| Very (level 3) |  | 0.53 | 0.28, 1.01 |  |
| Least/moderate (levels 1&2) |  | 0.41 | 0.19, 0.86 |  |
| Type of venous access | Implanted port |  |  | <0.001 |
| External tunneled catheter |  | 3.21 | 1.83, 5.65 | <0.001 |
| PICC line |  | 7.00 | 1.82, 26.94 | <0.01 |
| Peripheral IV |  | 0.73 | 0.11, 5.02 | 0.75 |
| ^††^ED clinical status |  |  |  |  |
| Maximum temperature, *continuous* | - | 1.04 | 0.97, 1.11 | 0.29 |
| Hypotension | No | 2.05 | 0.90, 4.66 | 0.09 |
| Tachycardia | No | 1.56 | 0.97, 2.52 | 0.07 |
| Tachypnea | No | 1.11 | 0.71, 1.74 | 0.64 |
| Chills or rigors | No | 2.35 | 1.15, 4.81 | 0.02 |
| *Initial WBC counts |  |  |  |  |
| Absolute monocyte count, *continuous* | - | 0.57 | 0.22, 1.49 | 0.25 |
| Absolute lymphocyte count, *continuous* | - | 0.74 | 0.49, 1.10 | 0.14 |
| Absolute neutrophil count, *continuous* | - | 0.99 | 0.92, 1.06 | 0.80 |
| ^¶^Neutropenic, yes | No | 2.47 | 1.56, 3.91 | <0.001 |
| Abbreviations: CNS: Central nervous system; HSCT: Hematopoietic stem cell transplantation; PICC: Peripherally inserted central catheter; ED: Emergency department; WBC: White blood cell  *All laboratory values obtained within first two hours of ED arrival  ^†^Based on Intensity of Treatment Rating criteria (Kazak, et al. *Pediatric Blood & Cancer,* 2012)  ^††^Hypotension, tachycardia, and tachypnea refer to presence of age-based vital sign abnormalities within two hours of ED arrival  ^¶^Neutropenia defined as absolute neutrophil count <0.5 x10^3^/μL | | | | |

| Supplemental Table 4: Association of initial lactate level with secondary outcomes pertaining to illness severity | | | |
| --- | --- | --- | --- |
| Outcome | **Odds ratio**  **(per unit increase in lactate)** | **95% CI** | **p value** |
| Hospital length of stay, days | 1.15 | 1.07, 1.24 | <0.001 |
| Need for PICU admission | 1.68 | 1.41, 2.00 | <0.001 |
| PICU length of stay, days | 1.21 | 1.04, 1.40 | 0.01 |
| Non-invasive infection | 0.87 | 0.73, 1.02 | 0.094 |

| Supplemental Table 5: Encounter characteristics and outcomes for patients who were critically ill-appearing at initial ED presentation | |
| --- | --- |
|  | All encounters (n=21) |
| Characteristic | n (%) |
| Number of unique patients | 20 |
| Age in years, *median (IQR)* | 13.3 (3.7-16.0) |
| Sex |  |
| Female | 12 (57%) |
| Male | 9 (43%) |
| Underlying Diagnosis |  |
| Acute lymphoblastic leukemia | 6 (28%) |
| Acute myeloid leukemia | 2 (10%) |
| Lymphoma | 4 (19%) |
| Solid Tumor | 5 (24%) |
| CNS Tumor | 2 (9%) |
| Non-malignant hematologic disorder | 2 (10%) |
| Phase of therapy |  |
| ^†^On therapy | 20 (95%) |
| Off therapy within <6 months | 1 (5%) |
| ^††^Chemotherapy intensity |  |
| Most (level 4) | 4 (19%) |
| Very (level 3) | 12 (57%) |
| Least/moderate (levels 1&2) | 4 (19%) |
| HSCT within the last 6 months | 3 (14%) |
| ^¶^ED laboratory values |  |
| Lactate (mmol/L) | 3.2 (1.7, 5.1) |
| Absolute monocyte count (x10^3^/μL) | 0.10 (0.01, 0.67) |
| Absolute lymphocyte count (x10^3^/μL) | 0.33 (0.09, 0.77) |
| Absolute neutrophil count (x10^3^/μL) | 0.54 (0.01, 3.62) |
| ^§^Neutropenic, yes (n, %) | 10 (48%) |
| Outcomes | |
| One or more CDE within 48 hours | 20 (95%) |
| One or more IBI within 48 hours | 14 (67%) |
| Hospital LOS in days (median, IQR) | 12.2 (3.2, 17.1) |
| PICU admission, n (%) | 19 (90%) |
| PICU LOS in days (median, IQR) | 4.0 (1.0, 10.0) |
| 30-day all-cause mortality, n (%) | 5 (25%) |
| 30-day infection-related mortality, n (%) | 4 (20%) |
| Abbreviations: ED: Emergency department; IQR: Interquartile range; CNS: Central nervous system; HSCT: Hematopoietic stem cell transplantation; CDE: Clinical deterioration event; IBI: Invasive bacterial infection; LOS: Length of stay; PICU: Pediatric intensive care unit  ^†^Includes patients receiving chemotherapy or within 6 months of HSCT  ^††^Based on Intensity of Treatment Rating criteria (Kazak, et al. *Pediatric Blood & Cancer,* 2012)  ^¶^ Initial values within two hours of ED arrival (lactate in mmol/L and white blood cell counts) reported as median (IQR). Presence of neutropenia is reported as n (%)  ^§^Neutropenia defined as absolute neutrophil count <0.5 x10^3^/μL | |
